# Comparative analysis of bacterial diversity in two hot springs in Hefei, China

**DOI:** 10.1038/s41598-023-32853-5

**Published:** 2023-04-10

**Authors:** Feng-Qin Zhang, Jun Liu, Xiao-Ju Chen

**Affiliations:** 1 College of Chemistry and Material Engineering, Chaohu University, Chaohu, 238024 Anhui China; 2 College of Biology and Food Engineering, Anhui Polytechnic University, Wuhu, 241000 Anhui China

**Keywords:** Computational biology and bioinformatics, Ecology, Genetics, Microbiology, Molecular biology, Environmental sciences

## Abstract

Hot springs are extreme ecological environments of microbes. The study is the first comparative analysis of bacterial diversity of Tangchi and Bantang hot spring water samples collected in Hefei, China, which is conducive to the further development and utilization of microbial resources in hot springs. Illumina MiSeq system was utilized to sequence and analyze the bacterial 16S rRNA gene from hot spring water samples by bioinformatics, to probe into the bacterial abundance and diversity of two hot springs in Hefei. Results revealed that prevalent bacterial phyla in Tangchi hot spring were Bacillota and Aquificota, and the prevalent bacterial genus was *Hydrogenobacter*; prevalent phyla in Bantang hot spring were Pseudomonadota followed by Actinobacteriota, and prevalent genera were *CL500-29_marine_group* and *Polynucleobacter*. More species and higher evenness in Bantang hot spring than those in Tangchi hot spring. In MetaCyc pathway analysis, the major pathways of metabolism existed in the bacteria from the two hot springs were ‘pyruvate fermentation to isobutanol (engineered)’, ‘acetylene degradation’, ‘carbon fixation pathways in prokaryotes’, ‘nitrate reduction I (denitrification)’, ‘methanogenesis from acetate’, ‘superpathway of glucose and xylose degradation’, etc.

## Introduction

A hot spring is an underground spring, whose water temperature is higher than that of surrounding areas, and it is rich in microbial resources^1^. The hot spring environment is relatively similar to the earth's early environment, and its unique physical and chemical characteristics promote the formation of special microbial groups. Thermophilic bacteria suitable for extreme environmental conditions gather in the hot spring environment. They are important exploitable microbial resources^2^. A large number of taxonomic populations of thermophilic bacteria have been found in the United States, India, Turkey, etc.^3–5^. In recent years, China has also made many achievements in the field of hot spring microbial research. Some thermophiles have been separated and identified in many hot springs in Yunnan, Sichuan and Jiangxi^6–8^.

The famous Tan (city)–Lu (river) fault runs through the territory in a northeast direction. The special geological background makes Hefei rich in geothermal resources and have a long history of hot springs. Bantang and Tangchi hot springs in Hefei have a history of more than 2200 years. They have been developed and utilized for generations and have continued to this day. They have become one of the hot spring forming areas with early development time, long utilization time and rich cultural connotation in China.

Bantang hot spring is exposed in the Paleozoic Ordovician carbonate rocks, located in the secondary faults and Paleozoic folds of the Tanlu fault zone, and the water quality type is SO_4_-Ca. It has a radon content of 92.85 kBq/m^3^ and a fluorine content of 2.60 mg/L. According to the naming standard of mineral water, it can be called "radon mineral water" and "fluorine mineral water"^9^. However, Tangchi hot spring is located in Mesozoic volcanic rocks, and the water quality type is HCO_3_-Ca·Mg. Its fluorine content is 12.67 mg/L, which meets the naming standard of "fluorine water" for medical and mineral water^10^.

Although Hefei has a long history in the development of tourism, vacation, recuperation and health care resources of these two hot springs, there is little research on the development of their microbial resources. In this research, the 16S rRNA gene amplification sequencing technology was used to sequence the v3-v4 region of bacterial 16S rRNA gene from two hot springs through the high-throughput sequencing platform Illumina MiSeq PE300. The community composition and diversity characteristics of bacteria from the two hot springs in Hefei were analyzed and compared. The bacterial diversity of the two hot springs was studied for the first time, which will provide basic information for understanding the bacterial diversity of hot springs in Hefei. This study will establish a scientific basis for the further development and utilization of hot spring microbial resources in Hefei in the future, and it will be conducive to the development of microbial ecology in the hot spring environment.

## Materials and methods

### Sample collection and processing

Water samples were collected from two different hot springs located in Lujiang and Chaohu districts of Hefei, China: Tangchi hot spring (TangChi) (117.54° E, 31.38° N) and Bantang hot spring (BanTang) (117.54° E, 31.38° N) during April 2022. Tangchi hot spring and Bantang hot spring were constructed as an artesian tube and an artesian well respectively. Triplicate samples of 5 L collected from both sampling points were put into sterile bottles, and temperature and pH of samples were estimated on site. Back to the lab, triplicate samples were pooled and filtered by 0.22 μM sterile filter membrane to enrich microbes. Then, these filter membranes were preserved at − 80 °C for standby^7,11^.

### DNA isolation

Total community genome from the two water samples were isolated by an E.Z.N.A™ MagBind Soil DNA Kit (Omega, M5635-02, USA), according to the manufacturer's instructions. DNA integrity was checked with 0.8% agarose gel. The DNA concentration was detected by a Qubit 4.0 (Thermo, USA) to make certain of sufficient quantity of high-quality genome isolated.

### 16S rRNA gene amplification and library preparation

PCR was started promptly after the DNA was isolated. The v3–v4 hypervariable region amplification of the 16S rRNA gene of bacteria was performed with the two universal primers (PAGE purified) 341F (CCTACGGGNGGCWGCAG) and 805R (GACTACHVGGGTATCTAATCC)^12^. There were two round amplifications in this study. The first round amplification was set up with the following system: DNA template 10–20 ng; Bar- PCR forward primer (10 µM) 1 µL; PCR reverse primer (10 µM) 1 µL; 2 × Hieff^®^ Robust PCR Master Mix (Yeasen, 10105ES03, China) 15 µL (total 30 µL). PCR was run according to the procedure: first 1 cycle of denaturing at 94 °C for 3 min, then 5 cycles of denaturing at 94 °C for 30 s, annealing at 45 °C for 20 s, elongation at 65 °C for 30 s, afterwards 20 cycles of denaturing at 94 °C for 20 s, annealing at 55 °C for 20 s, elongation at 72 °C for 30 s and a final extension at 72 °C for 5 min. The second round amplification introducing Illumina bridge PCR compatible primers was set up with the following system: PCR products 20–30 ng; PCR forward primer (10 µM) 1 µL; Index-PCR reverse primer (10 µM) 1 µL; 2 × Hieff^®^ Robust PCR Master Mix (Yeasen, 10105ES03, China) 15 µL (total 30 µL). PCR was run according to the procedure: 1 cycle of denaturing at 95 °C for 3 min, then 5 cycles of denaturing at 94 °C for 20 s, annealing at 55 °C for 20 s, elongation at 72 °C for 30 s, and a final extension at 72 °C for 5 min. The final amplicon was detected by electrophoresis in 2% agarose gel, purified by Hieff NGS™ DNA Selection Beads (Yeasen, 10105ES03, China), quantified by a Qubit^®^ 4.0 Green double-stranded DNA assay and quality checked by a Bioanalyzer (Agilent 2100, USA). Eventually, the purified amplicon was pair-end sequenced by an Illumina MiSeq system (Illumina MiSeq, USA) at Sangon BioTech in China.

### ASV clustering, representative tags alignment and biological classification

Cutadapt software (version 1.18)^13^ was used to cut the adaptor and primer sequences for off-machine double-ended sequence data. The DADA2(version 1.14.0)^14^ package of R was used to filter, correct and denoise each sample sequence, and then the double-ended data were concatenated, and the chimera was identified and removed by the algorithm to obtain the ASV sequence (default parameter). The ASV table was constructed according to the occurrence times of ASV sequences in each sample. Based on the Silva database^15^ (version 138.1), using the RDP classifier (version 2.12)^16^ for species annotation, the species information of each ASV at different taxonomic levels can be obtained.

### Statistical analysis

The alpha diversity indexes (containing Chao, Simpson, and Shannon indexes) were quantified according to ASV richness and calculated using Mothur software (version 3.8.31)^17^. To estimate sample adequacy, rarefaction curve of ASV numbers was established. The rank abundance curve and rarefaction curve were made by ggplot2 package (version 3.3.5) in R software (version 3.6.0).

### Function prediction

To compare the bacterial 16S rRNA gene sequences from Tangchi and Bantang hot spring with the functional spectrum database MetaCyc analyzing biological metabolic pathway of known metabolic functions separately, metabolic functions were predicted using PICRUSt2 (version 2.4.1)^18^.

### Nucleotide sequence accession numbers

16S rRNA gene sequencing data were accepted at NCBI under the accession nos. SRR20710118 and SRR20831105.

## Results

### Information of water samples collected

The temperature and pH of Tangchi hot spring were tested as 63.9 ℃ and pH 8.2 (alkaline) on site. However, those of Bantang hot spring were 45.8 ℃ and pH 6.5 (weakly acidic).

### Sequence merging and assembling

**Figure 1:**
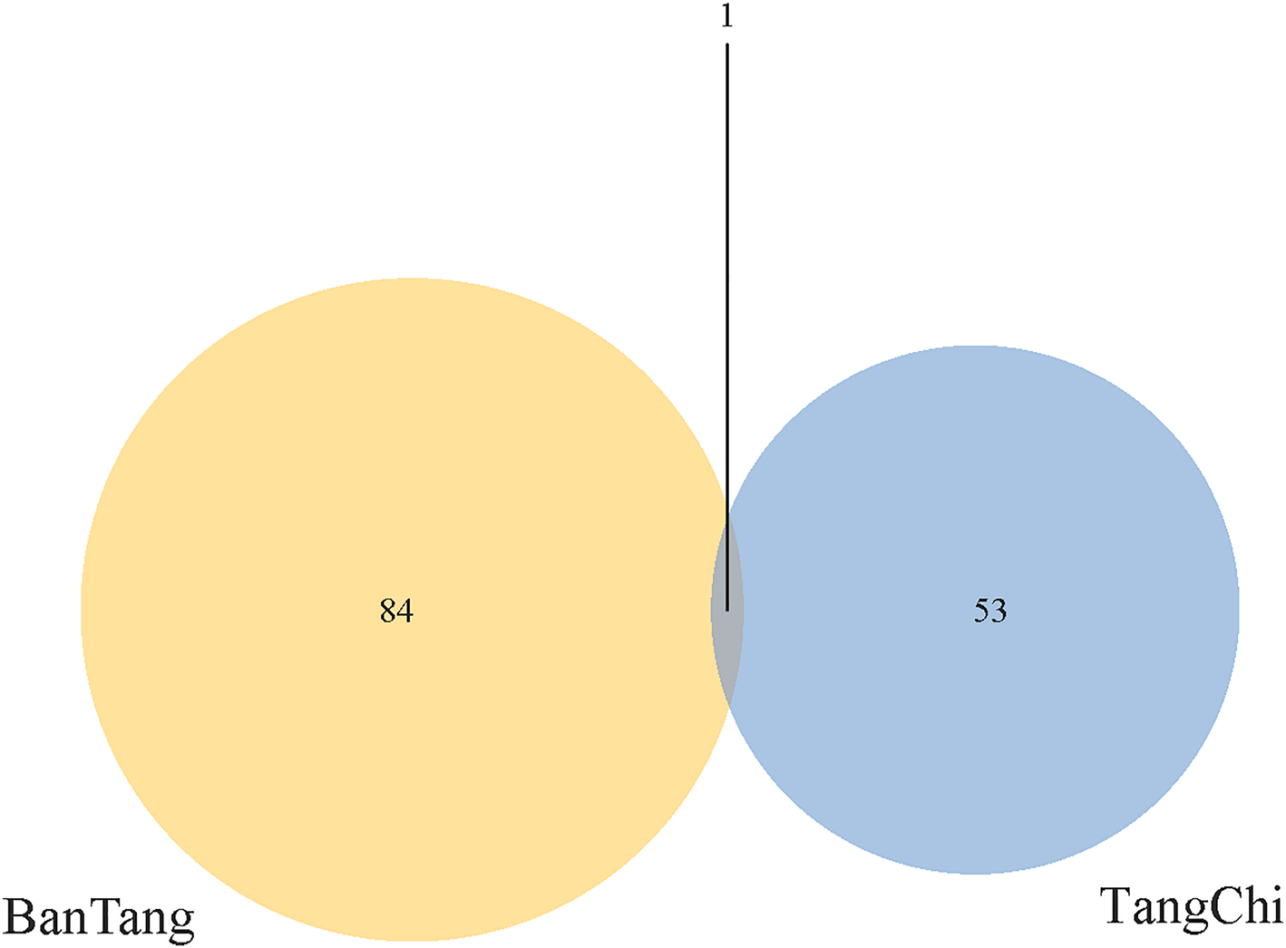
ASV numbers of bacterial communities from two hot springs.

After the hot spring sample sequences were split and removed redundancy, ASV clustering was carried out under100% similarity (Fig. 1). The results showed that 138 ASVs were received from two hot springs in all. The number of ASVs from Bantang hot spring (85) was more than that from Tangchi hot spring (54), and there was 1 ASV in common from the two hot springs, accounting for about 0.72% of the total ASVs.

### Bacterial community and abundance

The ASVs representative sequences of two hot springs were aligned and identified, and the community information of hot spring bacteria in 9 phyla, 13 classes, 23 orders, 29 families and 31 genera were obtained.

**Figure 2:**
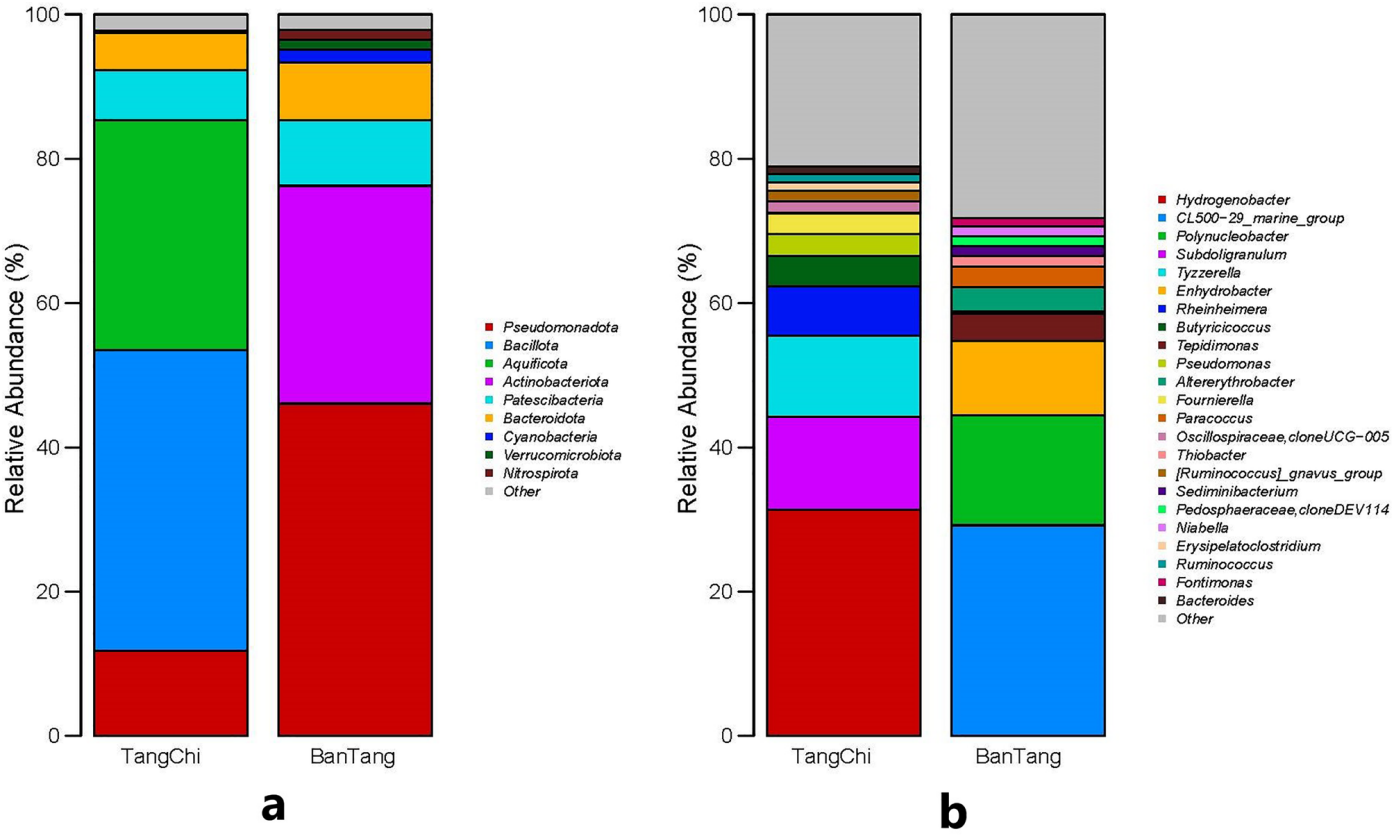
Phylum and Genus level distribution of bacterial community in two hot springs: (**a**) Phylum level; (**b**) Genus level.

At the phylum taxonomic level, bacteria with relative abundance less than 1% were attributed to others, and the composition of bacterial phyla from the two hot springs was significantly different. Tangchi hot spring bacteria mainly belonged to 5 groups, while Bantang hot spring bacteria mainly belonged to 7 groups (Fig. 2a). Bacterial communities from Tangchi hot spring were Bacillota (41.63%), Aquificota (31.84%), Pseudomonadota (11.82%), Patescibacteria (6.98%), Bacteroidota (5.16%). Bantang hot spring bacterial communities were Pseudomonadota (46.02%), Actinobacteriota (30.11%), Patescibacteria (9.07%), Bacteroidota (8.04%), Cyanobacteria (1.81%), Verrucomicrobiota (1.36%), Nitrospirota (1.29%). Pseudomonadota, Patescibacteria, Bacteroidota were discovered to be common bacterial phyla in two hot springs. However, Bacillota, Aquificota were in Tangchi hot spring, not in Bantang hot spring; Actinobacteriota, Cyanobacteria, Verrucomicrobiota, Nitrospirota were in Bantang hot spring, not in Tangchi hot spring.

At the genus taxonomic level, bacteria with relative abundance less than 1% and meaningless (i.e. uncultured and norank) were attributed to others, Tangchi hot spring bacteria belonged to 13 groups, and Bantang hot spring bacteria mainly belonged to 12 groups (Fig. 2b). Tangchi hot spring bacterial communities were *Hydrogenobacter* (31.38%), *Subdoligranulum* (12.80%), *Tyzzerella* (11.23%), *Rheinheimera* (6.87%), *Butyricicoccus* (4.22%), *Pseudomonas* (3.08%), *Fournierella* (2.87%), etc.; Bantang hot spring bacterial communities were *CL500-29_marine_group* (29.19%), *Polynucleobacter* (15.20%), *Enhydrobacter* (10.32%), *Tepidimonas* (3.78%), *Altererythrobacter* (3.38%), *Paracoccus* (2.80%), etc. There were no common bacterial genus in the two hot springs. Results suggested that bacterial genera composition of the two water samples were very different.

### Alpha diversity analysis of bacteria

#### Diversity index analysis

Alpha diversity indexes of bacteria from two hot springs were evaluated (Table 1). Shannon diversity index is utilized to estimate the microbial diversity in the sample. Shannon and Simpson indexes are often used for reflecting alpha diversity. The larger the Shannon value, the higher the bacterial diversity. Whereas the Simpson index is the opposite. Both Chao and Ace indexes are used for estimating ASV number in the community. Results suggested that Ace, Chao, Shannon and Simpson indexes of bacteria in Tangchi hot spring had obvious difference with those in Bantang hot spring. Ace, Chao and Shannon indexes of bacteria in Bantang hot spring were greater than those in Tangchi hot spring, but Simpson index of bacteria in Bantang hot spring was less than that in Tangchi hot spring, indicating that the bacterial diversity in Bantang hot spring (45.8 ℃) was higher than that in Tangchi hot spring (63.9 ℃). In other words, the higher the hot spring temperature, the level of bacterial diversity is relatively lower, which indicates that temperature is an important factor affecting the level of bacterial diversity in hot springs.

**Table 1:** Bacterial alpha diversity indices of the two hot springs.

Sample	Shannon	Chao	Ace	Simpson
TangChi	2.968949	55	55	0.085989
BanTang	3.392597	85	85	0.062225

#### Diversity curve analysis

**Figure 3:**
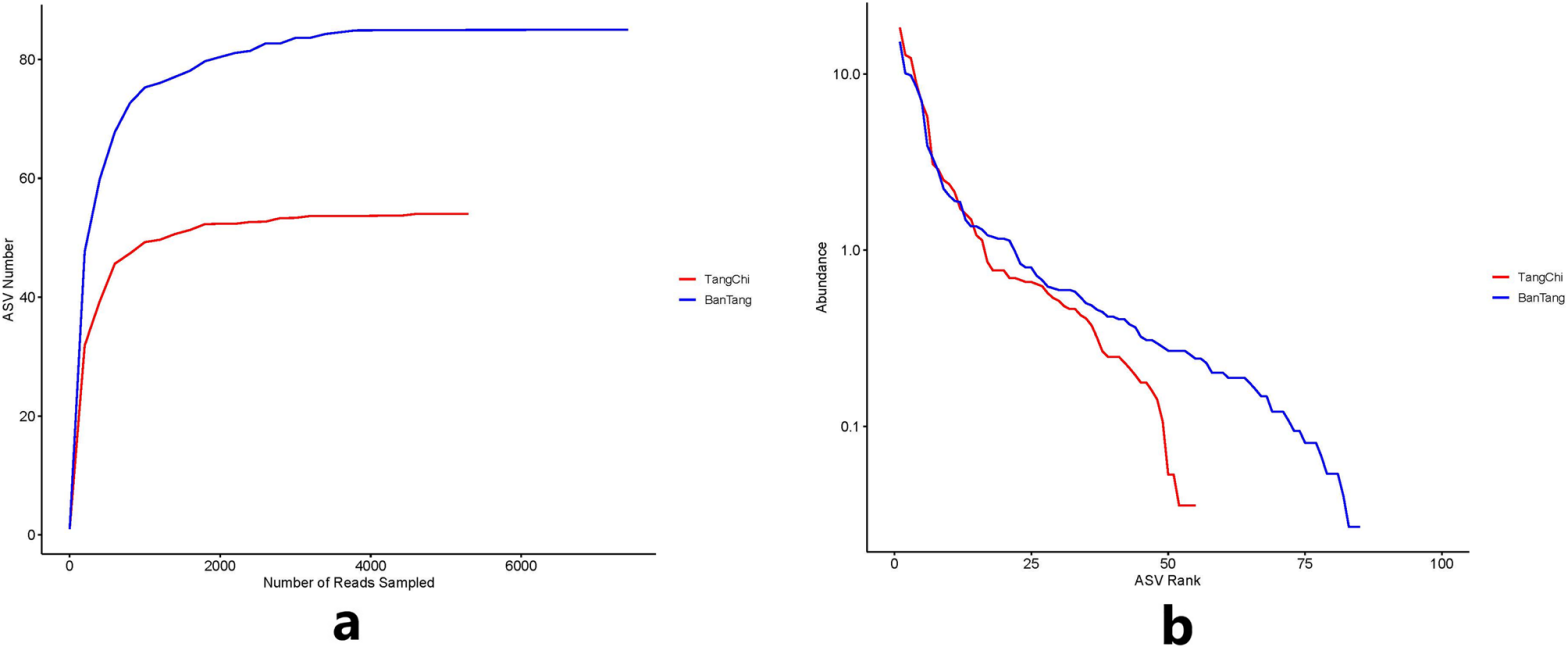
α-diversity comparison: (**a**) Rarefaction curve; (**b**) Rank abundance curve.

The ASV rarefaction curve and rank abundance curve of the two hot springs were plotted (Fig. 3a,b).Whether the amount of sequencing data is sufficient can be judged according to whether the rarefaction curve is gentle. The increase rate of ASV number gradually flattened along with the increase of the number of sampling sequences of Tangchi and Bantang hot springs, and the curve tended to be horizontal relative to the x-axis, especially rarefaction curve of Tangchi hot spring was flatter. It showed that the sequencing depth of two hot spring samples was reasonable. Rank abundance curve is used for explaining two aspects of sample diversity (that is, the richness and evenness) at the same time. The richness of bacteria is represented by the length of the curve on the horizontal axis. The wider the curve, the higher the richness of bacteria. Meanwhile, the uniformity of bacterial composition is represented by the shape of the curve. The flatter the curve, the higher the uniformity of bacterial composition. Results showed that more richness and higher evenness in Bantang hot spring than those in Tangchi hot spring.

#### Prediction of microbial functional gene

**Figure 4:**
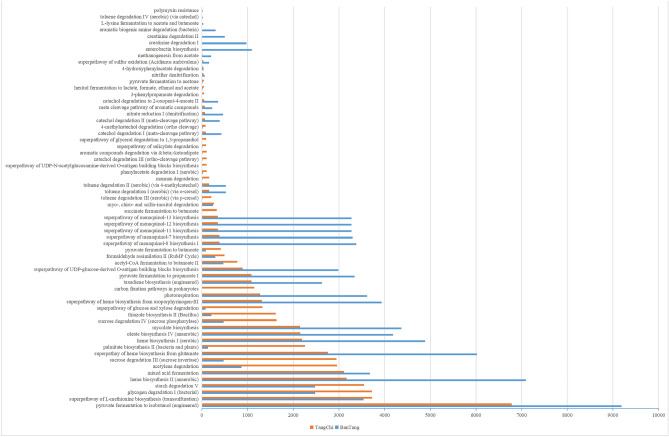
Part of MetaCyc pathway predicting functions from the 16S rRNA gene sequences of the two hot springs.

According to the prediction results, the annotation corresponding to each functional spectrum database of each sample was acquired. In MetaCyc pathway analysis, the major pathways of metabolism as ‘pyruvate fermentation to isobutanol (engineered)’, ‘acetylene degradation’, ‘carbon fixation pathways in prokaryotes’, ‘nitrate reduction I (denitrification)’, ‘methanogenesis from acetate’, ‘superpathway of glucose and xylose degradation’, etc., were picked to show (Fig. 4). Among them, ‘carbon fixation pathways in prokaryotes’, ‘succinate fermentation to butanoate’, ‘superpathway of UDP-*N*-acetylglucosamine-derived O-antigen building blocks biosynthesis’, ‘superpathway of glycerol degradation to 1,3-propanediol’ and ‘3-phenylpropanoate degradation’ were only possessed by bacteria in Tangchi hot spring. However, ‘enterobactin biosynthesis’, ‘creatinine degradation I’, ‘creatinine degradation II’, ‘aromatic biogenic amine degradation (bacteria)’, ‘l-lysine fermentation to acetate and butanoate’, ‘toluene degradation IV (aerobic) (via catechol)’ and ‘polymyxin resistance’ existed only in the bacteria from Bantang hot spring. Among the degradation pathways, enzymes mapped on xenobiotic degradation pathways generally belong to the classes of oxidoreductases, lyases and transferases^19^.

## Discussion

The results of the study provide a detailed comparative analysis of the bacterial community composition of Tangchi hot spring and Bantang hot spring in Hefei using 16S rRNA gene-based high throughput sequencing approach. The differences in alpha diversity and the composition of the bacterial community between the two hot springs may be related to the combination of number of environment factors^20^.

The prevalent phyla found in Tangchi hot spring were Bacillota (41.63%) and Aquificota (31.84%).They have also been reported in many moderately alkaline hot springs^21–23^. Aquificota belongs to eubacteria, but it is the closest to archaea and eukaryotes, which is a kind of rod-shaped bacteria living in moderate or ultra-high temperature environment^24^. The prevalent bacteria genus in Tangchi hot spring was mainly *Hydrogenobacter* (31.38%). A large number of this genus (abundance more than 30%) were detected in several hot springs in Rehai and Ruidian, Tengchong, Yunnan^25^. They are hydrogenoxidizing and obliterate chemolithoautotrophic bacteria^26^. Their energy metabolism mode in medium alkaline hot springs may be chemical autotrophy, that is, to obtain energy through the oxidation of H_2_^27^. Moreover, the enzyme pNAR (dissimilarity nitrate reductase) previously found only in archaea was discovered in *Hydrogenobacter* by Masafumi et al.^28^.

The prevalent bacterial phyla in Bantang hot spring were Pseudomonadota (46.02%) and Actinobacteriota (30.11%), which also have high abundance in Tibetan hot springs^29^ and Unnai hot spring^30^. Pseudomonadota is phototrophic bacterium as the second largest phylum of hydrogenogenic CO oxidizers^31^, and is one of the largest and most phenotypic branches of domain bacteria^32^. At the moment, the phylum ‘Pseudomonadota’ can degrade methyl tert-butyl ether (MTBE) polluting underground waters^33^. Actinobacteriota, one of the largest phyla in domain bacteria, are world-wide organisms and can live in a range of ecological environments. Because of their ubiquitous nature, they play some crucial roles to recycle substances, synthesize bioactive molecules and degrade complex polymers. They can produce a large number of naturally derived modern antibiotics and many other antifungal, antiparasitic, anticancer, antitumor and immunomodulator compounds. In addition, they are used in various biotechnology for producing many industrially important enzymes, organic acids, amino acids, pigments, vitamins and toxins. Therefore, as a result of great diversity and survivability under severe exogenous stress, Actinobacteriota are also used for ecosystem transformation, biotransformation and bioremediation programs^34^. The prevalent bacterial genus in Bantang hot spring were mainly *CL500-29_marine_group* (29.19%) and *Polynucleobacter* (15.20%), etc. To the best of our knowledge, *CL500-29_marine_group* and *Polynucleobacter* are both found in freshwater systems^35,36^, but not found in other hot springs yet.

In addition to Pseudomonadota, we found other photosynthetic bacteria in the two hot springs. Cyanobacteria was detected in Bantang hot spring. Cyanobacteria was reported by previous workers also in several hot springs in Tapovan hot spring^37^, Rupite hot spring^38^, and two alkaline hot springs in Yellowstone National Park^39^. Phototrophic bacteria present in hot springs may propel the entire microbial ecosystem.

In the previous study, strain LJTC-1^40^ (NCBI accession number: KT454966) and LJTC-2^41^ (NCBI accession number: OP132549) were obtained from Tangchi hot spring through separation and purification, belonging to *Thermoactinomyces* sp. and *Geobacillus* sp. Separately. CHBT-1721^42^ (NCBI accession number: KJ524642) was isolated from Bantang hot spring, belonging to *Bacillus* sp. And these strains all belonged to the phylum Bacillota. Among the high-throughput sequencing results, the bacteria in the phylum Pseudomonadota, Aquificota and Actinobacteriota were not isolated by culturable methods. There are many factors that limit the cultivability of microorganisms, mainly including substrate and growth conditions, recovery from dormancy, symbiotic interdependence, physical contact or spatial proximity, environmental physicochemical conditions, low abundance and competition, etc.^43^.

Microbes in different hot springs show different microbial community structures and functions^44^. According to gene function annotation, in MetaCyc pathway analysis, ‘carbon fixation pathways in prokaryotes’ was only found in Tangchi hot spring. Carbon dioxide is a greenhouse gas. Microbes with autotrophic metabolism assimilate inorganic carbon into organic carbon, making carbon unavailable to other organisms a core component of the global carbon cycle. With the rapid development of biochemical engineering and genetic engineering technology, the biological capture, transformation and utilization of carbon dioxide have made rapid progress as value-added products^45^. Tan et al.^46^ directly synthesized a degradable plastic PLA from carbon dioxide for the first time in the world, using a combination strategy of metabolic engineering and high-density culture on a light driven cyanobacteria platform. Carbon fixation can not only slow down the greenhouse effect, but also realize non grain fermentation^47^. The abundance of ‘nitrate reduction I (denitrification)’ and ‘superpathway of glucose and xylose degradation’ were higher in Bantang hot spring than that in Tangchi hot spring. Denitrification is an essential part of most wastewater treatment systems. Biological nitrogen removal has been widely concerned for its high cost-effectiveness, simple process and no secondary pollution^48^. ‘Superpathway of glucose and xylose degradation’ was very important metabolic pathway. Glucose and xylose are the main components of lignocellulose hydrolysate^49^. Moreover, lignocellulosic biomass is a renewable resource with abundant reserves, which has broad application prospects in the fields of energy^50^ and chemical industry^51^ through microbial fermentation. It can be seen that the bacteria in the two hot springs are of great value in environmental protection and industrial application.

## Conclusion

In this study, 16S rRNA high-throughput sequencing technology was used for the first time to compare and analyze the bacterial diversity of Tangchi hot spring and Bantang hot spring in Hefei, China. Results showed that the two hot springs in Hefei were rich in microbial resources. The bacterial community and abundance of Bantang hot spring are greater than those of Tangchi hot spring, and the prevalent bacterial phyla and prevalent bacterial genus in the two hot springs are significantly different, which supplies a theoretical basis for further exploring impact factors of microbial diversity in the later stage.

## Data Availability

16S rRNA gene sequencing data have been available at NCBI under the accession numbers: SRR20710118 (https://www.ncbi.nlm.nih.gov/sra/?term=SRR20710118) and SRR20831105 (https://www.ncbi.nlm.nih.gov/sra/?term=SRR20831105). Strain DNA sequences have been available at GenBank under the accession numbers: KT454966 (https://www.ncbi.nlm.nih.gov/nuccore/KT454966), OP132549 (https://www.ncbi.nlm.nih.gov/nuccore/OP132549) and KJ524642 (https://www.ncbi.nlm.nih.gov/nuccore/KJ524642).
